# Hydrophilic Aromatic Residue and *in silico* Structure for Carbohydrate Binding Module

**DOI:** 10.1371/journal.pone.0024814

**Published:** 2011-09-22

**Authors:** Wei-Yao Chou, Tun-Wen Pai, Ting-Ying Jiang, Wei-I Chou, Chuan-Yi Tang, Margaret Dah-Tsyr Chang

**Affiliations:** 1 Department of Computer Science, National Tsing Hua University, Hsinchu, Taiwan, Republic of China; 2 Institute of Molecular and Cellular Biology and Department of Medical Science, National Tsing Hua University, Hsinchu, Taiwan, Republic of China; 3 Department of Computer Science and Engineering and Center of Excellence for Marine Bioenvironment and Biotechnology, National Taiwan Ocean University, Keelung, Taiwan, Republic of China; 4 Simpson Biotech Co., Ltd, Taoyuan County, Taiwan, Republic of China; Griffith University, Australia

## Abstract

Carbohydrate binding modules (CBMs) are found in polysaccharide-targeting enzymes and increase catalytic efficiency. Because only a relatively small number of CBM structures have been solved, computational modeling represents an alternative approach in conjunction with experimental assessment of CBM functionality and ligand-binding properties. An accurate target-template sequence alignment is the crucial step during homology modeling. However, low sequence identities between target/template sequences can be a major bottleneck. We therefore incorporated the predicted hydrophilic aromatic residues (HARs) and secondary structure elements into our feature-incorporated alignment (FIA) algorithm to increase CBM alignment accuracy. An alignment performance comparison for FIA and six others was made, and the greatest average sequence identities and similarities were achieved by FIA. In addition, structure models were built for 817 representative CBMs. Our models possessed the smallest average surface-potential *z* scores. Besides, a large true positive value for liagnd-binding aromatic residue prediction was obtained by HAR identification. Finally, the pre-simulated CBM structures have been deposited in the Database of Simulated CBM structures (DS-CBMs). The web service is publicly available at http://dscbm.life.nthu.edu.tw/ and http://dscbm.cs.ntou.edu.tw/.

## Introduction

Carbohydrate-binding modules (CBMs) are structural domains found within polysaccharide-targeting enzymes but do not contain the active sites. CBMs increase the catalytic efficiencies of their enzymes by bringing the catalytic sites into prolonged and intimate contact with substrates [1], [2]. Currently, CBMs are found among 64 protein families which are defined in CAZy, a regularly updated database (http://www.cazy.org/) [3], according to their homologies and functionalities. In addition to conventional carbohydrate-binding functions, CBMs have been reported to participate in an immune system-related allergic reaction [4]. Historically, several binding modules that were found to bind cellulose were named cellulose-binding domains (CBDs) [5], [6]. With the subsequent identification of CBMs that bind a wide range of polysaccharides, including crystalline cellulose, hemicelluloses, such as glucan, xylan, mannan, and glucomannan as well as insoluble and soluble starches [7], the generalized term CBM has evolved. CBMs represent all of the non-catalytic sugar-binding modules derived from glycoside hydrolases. Furthermore, ligand-binding site properties and structural topologies for CBMs have been summarized and reviewed [8]. CBMs are classified into three types in terms of ligand-binding function: surface-binding (type A), glycan-chain binding (type B) and small-sugar binding (type C) [9]. Type A CBMs possess a platform-like or horizontal hydrophobic surface consisting of aromatic residues. The planar conformation of the type A binding site interacts with the flat surfaces of crystalline polysaccharides. The binding site architecture of type B CBMs form a cleft or groove shape in which aromatic residues interact with free single polysaccharide chains. Type C CBMs are characterized by steric restrictions in the binding site and only binds mono-, di-, or tri-saccharides. Regardless of the three types, aromatic residues contribute to stacking interactions with the sugar rings leading to van der Waals interactions and the side chain of polar residues may provide hydrogen bonds with the sugar ligand [10]. Despite of their low sequence identity, CBMs are structurally characterized by a β-sandwich fold and seven fold types have been observed: β-sandwich, β-trefoil, cysteine knot, unique, OB fold, hevein fold and hevein-like fold [7]. Among these seven, β-sandwich and β-trefoil foldings are found in most CBM families. The best studied system to date are the starch-binding CBMs whose structural, functional and evolutionary relationships has been analyzed based on the comprehensive understanding of CBM20 [11]. Starch-binding CBMs have been identified in ten CBM families (20, 21, 25, 26, 34, 41, 45, 48, 53 and 58).

X-ray crystallography, nuclear magnetic resonance (NMR) spectroscopy, and electron microscopy (EM) have been used to determine the three-dimensional (3D) structures of CBMs [12], [13], [14]. In general, a 3D structure reveals certain of the chemical and physical characteristics of a protein, which can increase our knowledge of substrate-protein, ligand-protein, and/or protein-protein interactions and structural folding motifs. Usually, hydrophilic residues are found on protein surfaces and are the residues that interact with substrates, ligands, or other proteins. Conversely, hydrophobic residues are usually located in the core of a protein and stabilize the structure. Given the spatial coordinates of a protein, *in silico* investigations, e.g., molecular docking, can be performed before attempting more timely, costly, and labor intensive “wet” experiments [15]. However, as of August, 2011, more than 16 million sequences were available in the UniProtKB/TrEMBL database (http://www.ebi.ac.uk/uniprot/TrEMBLstats/), whereas fewer than 76 thousand 3D structures had been deposited in the Protein Data Bank (http://www.rcsb.org/). Additionally, the number of sequenced proteins is increasing more rapidly than is the number of solved structures. Although the technical aspects of the methods used to determine 3D protein structures have substantially improved over the years and grown more sophisticated, their execution remains expensive and time-consuming. For protein structures that have not been solved experimentally, *in silico* modeling, e.g., homology modeling [16], fold recognition [17], and *ab initio* prediction [18] can be used instead. Of these three approaches, homology modeling is the most accurate [19] and it involves three major steps: template selection, target-template sequence alignment, and model building. In practice, the target-template sequence alignment and template selection are the most critical steps for accurate homology modeling. 30% sequence identity is the minimum percentage that is necessary for accurate homology modeling [16], because accurate target-template sequence alignment is sensitive to high sequence identities. Less than 5% of CBMs has experimentally solved structures, and the sequence identities among families are usually less than 30%. Fortunately however, CBM family members have similar secondary structures and conserved potential solvent-accessible aromatic residues, which we refer to as hydrophilic aromatic residues (HARs), which are often responsible for ligand-binding function. The prototype of this idea was successfully applied to predict an *in silico* structure for *Rhizopus oryzae* glucoamylase (CBM21) in low sequence identity condition and the predicted ligand-binding residues were experimentally verified [20]. When the positions of the conserved HARs and the secondary structure elements of CBMs were integrated into a feature-incorporated alignment (FIA) algorithm [21], we found an ∼5% improvement in the average sequence similarity and identity compared with target-template alignments obtained using six leading alignment algorithms. The improved alignments were used to identify conserved ligand-binding aromatic residues in CBM domains for which 3D structures were unavailable. For the study reported herein, we were dedicated to construct *in silico* structures for CBMs referred as targets. Therefore, 93 non-redundant experimentally determined CBM structures and 817 representative CBM sequences for which the corresponding structures have yet to be solved were used as templates and targets, respectively. A template filter algorithm was developed to rank the likelihood that a template structure would be a good match for a given target by assessing the proposed identity level and similarity level of the template-target sequence alignments produced by the FIA algorithm in the preceding step (the proposed identity level and similarity level are defined in Materials and Methods). Then, *in silico* structures were built using single-template and combinations of double templates. Finally, for each target the best *in silico* structure was identified according to its surface-potential *z* score, which was the lowest among the structures

## Results

### Functional correlation

**Figure 1:**
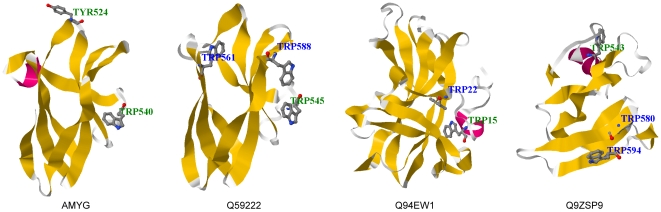
CBMs containing known ligand-binding aromatic residues and their *in silico* structures. The four selected CBMs with experimentally determined ligand-binding aromatic residues are *Aspergillus oryzae* glucoamylase (AMYG, CBM20, [22]), *Bacillus sp.* TS-23 α-amylase (Q59222, CBM20, [23]), *Nicotiana tabacum* Nictaba (Q94EW1, CBM22, [24]), and *Solanum lycopersicum* endo-β-1,4-D-glucanase (Q9ZSP9, CBM49, [25]). Known ligand-binding aromatic residues are highlighted in ball and stick model. HARs and non-HARs are texted in green and blue, respectively. Five out of ten known ligand-binding residues are predicted as HARs. The 3D structures were rendered by Jmol (http://www.jmol.org/).

The goal of this work reported herein was to establish reliable *in silico* structures for CBMs by improving the target-template sequence alignment procedure and template filter steps. In addition, our long-range goal is to apply the results of our *in silico* models to biological applications that involve CBMs. Table 1 contained the profile information for four CBMs from different families and their identified HARs. Figure 1 showed that known ligand-binding aromatic residues Tyr^524^ and Trp^540^ in CBM20 *Aspergillus oryzae* glucoamylase [22]; Trp^545^, Trp^561^ and Typ^588^ in CBM20 *Bacillus sp.* TS-23 α-amylase [23]; Trp^15^ and Trp^22^ in CBM22 *Nicotiana tabacum* Nictaba [24]; and Trp^543^, Trp^580^ and Trp^594^ in CBM49 *Solanum lycopersicum* endo-β-1,4-D-glucanase [25] have been experimentally validated, even though the corresponding *in vitro* structures are not available. Among ten known ligand-binding aromatic residues highlighted in ball and stick, five colored in green in Figure 1 were predicted as HARs with a 50% true positive rate. The lower surface-potential *z* score indicated that the structures are more stabilized and reliable. These four *in silico* structures each possessed low surface-potential *z* score for the CBM and the known ligand-binding HARs were located on the surfaces of the *in silico* CBM structures.

**Table 1:** Profile summaries for selected CBMs containing known ligand-binding aromatic residues.

Family	UniProt	Protein	Position	Organism	HARs	Template(s)	Identity	*Z* score
CBM20	AMYG	Glucoamylase	511–606	*Aspergillus oryzae*	**Y524**, **W540**, Y553, W560, W586	1pam–1d3c	39.1	–0.545
CBM20	Q59222	α-amylase	515–608	*Bacillus sp.*TS-23	**W545**, Y558, Y606	1cyg–1ac0	50.5	–0.838
CBM22	Q94EW1	Nictaba	1–165	*Nicotiana* *tabacum*	W5, **W15**, Y45, Y59, F99, W121, F129, F130, W151, F160	1dyo	30.1	0.445
CBM49	Q9ZSP9	Endo-β-1,4-D-glucanase	529–625	*Solanum* *lycopersicum*	**W543**, Y585, F589, Y622	2j1v–2orz	31.4	–0.186

The bold fonts indicate experimentally determined ligand-binding residues. No experimental data concerning their ligand-binding abilities is available for the unannotated HARs. The used template structures are 1pam [39], 1d3c [40], 1cyg (*N.A.*), 1ac0 [41], 1dyo [42], 2j1v [24] and 2orz [43].

### Prediction analysis

**Figure 2:**
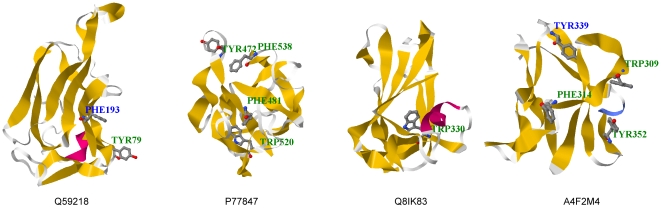
CBMs containing aromatic residues conserved to known ligand-binding residues and their *in silico* structures. The four selected CBMs with aromatic residues conserved to known ligand-binding residues are *Bacteroides ovatus* arabinosidase (Q59218, CBM4), *Caldocellum saccharolyticum* β-1,4-annanase (P77847, CBM6), *Plasmodium falciparum* LCCL domain-containing protein (Q8IK83, CBM32), and *Phaseolus vulgaris* starch synthase III (A4F2M4, CBM53). Known ligand-binding aromatic residues are highlighted in ball and stick model. HARs and non-HARs are texted in green and blue, respectively. Nine out of eleven aromatic residues conserved to known ligand-binding residues are predicted as HARs. The 3D structures were rendered by Jmol (http://www.jmol.org/).

**Figure 3:**
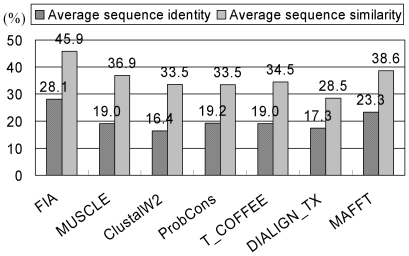
Average sequence identities and similarities for CBMs from seven sequence alignment methods. The sequence identities (similarities) are averaged from 75,981 (817 * 93) target-template pairwise sequence alignments. The used alignment programs are FIA [21], MUSCLE [32], ClustalW2 [33], ProbCons [36], T-COFFEE [35], DIALIGN-TX [34], and MAFFT [37].

We could use the locations of the known ligand-binding residues in the template structures to further characterize the ligand-binding functions with respect to conserved HARs in *in silico* structures. Table 2 listed four selected CBMs from different CBM families for which neither ligand-binding abilities nor *in vitro* structures have been experimentally determined. The aromatic residues conserved to reported ligand-binding aromatic residues were texted in green in Figure 2. (An additional sixteen examples are given in Supporting Information Table S1 and Figure S1). Tyr^79^ in *Bacteroides ovatus* arabinosidase (CBM4), Tyr^472^, Phe^481^, Trp^520^ and Phe^538^ in *Caldocellum saccharolyticum* β-1,4-mannanase (CBM6), Trp^330^ in *Plasmodium falciparum* LCCL domain-containing protein (CBM32), and Trp^309^, Phe^314^ and Tyr^352^ in *Phaseolus vulgaris* starch synthase III (CBM53) were identified as HARs positioned on *in silico* structural surface, indicating that they could be potential ligand-binding residues. Nine of eleven conserved aromatic residues were identified as HARs, hence a true positive rate of 81.8% was achieved. Notably, the average sequence identity and similarity obtained using FIA were greater than those obtained using the other alignment algorithms (see Figure 3). Moreover, none of their target-template alignments had sequence identities >28%, a value which is found for the most difficult examples of homology modeling [16]. Even though the sequence identities were always <30%, the predicted HARs were conserved in the alignments and located on the surfaces of the *in silico* structures. Our *in silico* structures support the idea that these predicted HARs can be potential ligand-binding residues.

**Table 2:** Profile summaries for selected CBMs containing aromatic residues conserved to known ligand-binding residues.

Family	UniProt	Protein	Position	Organism	HARs	Template(s)	Identity	*Z* score
CBM4	Q59218	Arabinosidase	59–197	*Bacteroides ovatus*	W76, *Y79*, F116, F117	2zex	26.9	–0.463
CBM6	P77847	β-1,4-mannanase	453–575	*Caldocellum saccharolyticum*	W461, *Y472*, Y477, *F481*, Y499, Y514, *W520*, *F538*, Y564	1w9s–1uxx	27.9	–0.454
CBM32	Q8IK83	LCCL domain-containing protein	305–420	*Plasmodium falciparum*	Y307, Y329, *W330*, F335, Y339, F380	2j1v–2j7m	26.7	–0.510
CBM53	A4F2M4	Starch synthase III	280–365	*Phaseolus vulgaris*	F306, *W309*, *F314*, W327, F346, *Y352*	2v8l–2c3w	24.3	–0.367

The italics denote aromatic residues conserved to reported ligand-binding residues in corresponding template(s). No experimental data concerning their ligand-binding abilities is available for the unannotated HARs. The used template structures are 2zex [44], 1w9s [45], 1uxx [46], 2j1v [24], 2j7m [47] and 2v8l [48], 2c3w [49].

### Performance comparison

**Figure 4:**
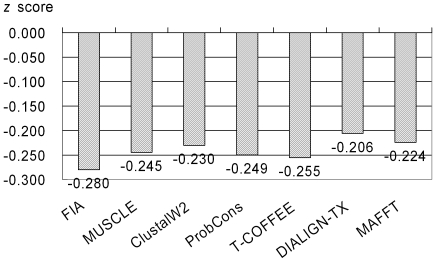
Average *z* scores for *in silico* structures built based on seven sequence alignment methods. The *z* scores are averaged from the lowest *z* scores of top five *in silico* structures for each target CBM. The used alignment programs are FIA [21], MUSCLE [32], ClustalW2 [33], ProbCons [36], T-COFFEE [35], DIALIGN-TX [34], and MAFFT [37]. The surface potential *z* scores are evaluated by PROSA2003 [38].

**Figure 5:**
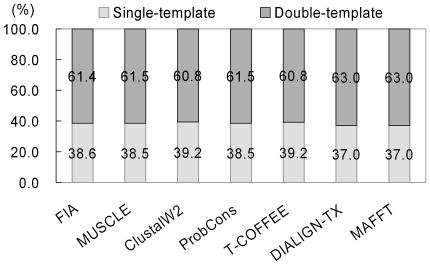
Percentages of structures built using single- and double-templates based on seven sequence alignment methods. The percentages indicate the top five structures with lowest *z* scores for each target CBM are derived from either single-template or double-template homology modeling. The used alignment programs are FIA [21], MUSCLE [32], ClustalW2 [33], ProbCons [36], T-COFFEE [35], DIALIGN-TX [34], and MAFFT [37].

The alignment performances for FIA and the six leading alignment programs were compared. Figure 3 showed the average number of sequence similarities and identities for the FIA, MUSCLE, ClustalW2, ProbCons, T-COFFEE, DIALIGN-TX, and MAFFT alignments. Each of the 817 target sequences was individually aligned with each of the 93 template sequences for a total of 75,981 (817 * 93) target-template sequence alignments. For the FIA alignments, the greatest average sequence similarity (45.9%) and average sequence identity (28.1%) were found, whereas the smallest average sequence similarity (28.5%) was found for the DIALIGN-TX alignments, and the smallest average sequence identity (16.4%) was found for the ClustalW2 alignments. Figure 4 plotted the average *z* scores for the *in silico* structures derived from alignments of various alignment tools. The modeling procedures used to produce the *in silico* structures were identical except for the target-template sequence alignments, which used different alignment tools. The *z* scores reported in Figure 4 were the average of the five smallest *z* scores for each target CBM. For FIA, the smallest average *z* score is –0.280, whereas that for DIALIGN-TX is –0.206. The relative numbers (as percentages) of structures build using single templates and double templates were given in Figure 5. For the *in silico* structures, between 60.8% and 63.0% of the top five candidates were derived from double-template modeling indicating that double-template modeling produced smaller *z* scores than the single-template approach. In summary, the performance of FIA was superior for both sequence alignment and structure building. The inclusions of conserved secondary structure elements and HAR positions in FIA improved model building and identification of ligand-binding residues.

## Discussion

### Template choice

We expected that multiple-template-based structure modeling would increase the quality of the models. The average *z*-scores in Figure 4 showed that simulated structures based on double-template ranked in the top five were at least 60%, indicating that double-template modeling produces more accurate structural predictions. An assessment of the raw data, which are not shown, indicates that double-template-based modeling is slightly better than is the single-template-based approach. Specifically, the best combinations of double templates usually consisted of one major template sequence that matched the target sequence as main skeleton and a second compatible or complementary sequence. “Compatible” indicates that the two templates are homologues with few insertions and deletions in their aligned sequences, whereas “complementary” denotes that one template includes an aligned sequence that is mismatched in the other template-target sequence alignment. Therefore, a key factor for successful homology modeling is the ability to choose compatible or complementary combinations of templates rather than considering as many templates as possible, i.e., the use of two or more dissimilar templates may decrease the modeling quality. Another disadvantage associated with multiple-template-based homology modeling is the greater computational time as more combinations of template structures must be matched to the target and evaluated.

### Correlation of hydrophilic aromatic residue

**Figure 6:**
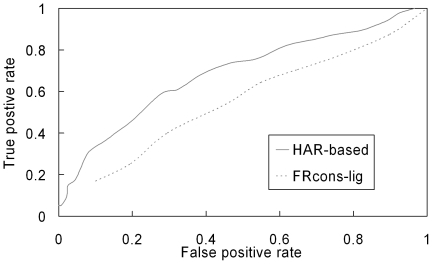
Receiver operating characteristic (ROC) curves of ligand-binding residue prediction. 97 known ligand-binding aromatic residues and 558 aromatic residues without experimental ligand-binding abilities in 49 template structures (see Supporting Information Table S2) are as positive and negative sets for prediction of ligand-binding aromatic residues, respectively. The ROC curves are generated by HAR-based and FRcons-lig [26] predictions.

Given that aromatic residues are known to participate in ligand-binding function in CBMs, the identification of HARs was introduced into our homology-modeling scheme as they could serve ligand-binding aromatic residues. The occurrence times for the two upstream and downstream polar residues flanking 97 known ligand-binding aromatic residues were determined (see Table 3). In addition to the polar residues, the occurrence times for glycine and alanine were also determined. The large occurrence times for glycine and alanine can be rationalized on the basis of their relatively small sizes, which would minimize steric conflicts with their neighboring aromatic residues. Moreover, the HAR identification procedure can be used as a simple but effective sequence-based ligand-binding residue predictor. 97 known ligand-binding aromatic residues and 558 aromatic residues without experimental ligand-binding abilities in 49 CBM structure templates were used as positive and negative sets, respectively (see Supporting Information Table S2). In comparison with a sequence-based ligand-binding residue predictor, FRcons-lig [26], which combined information of amino acid conservation, secondary structure and relative solvent accessibility, the number of true positives for any given number of false positives was larger for HAR-based predictor than FRcons-lig as shown in Figure 6. With the cut-off threshold for the sum of weighted scores set to 97, the true positive and false positive percentages were 74.2% and 46.2%, respectively. Because some of the “real” ligand-binding aromatic residues have yet to be determined, the false and true positive percentage is an overestimation and underestimation, respectively. When more experimentally determined ligand-binding aromatic residues become available, the false and true positive percentage are expected to be decreased and increased, respectively. Furthermore, our intention was not just to build reliable *in silico* structures but also to be able to correlate structural aspects of CBMs with the corresponding experimental functional assays. Here, we mainly focused on the prediction of the HARs of CBMs that possibly bind substrate polysaccharides. In fact, a typical CBM ligand-binding site contains multiple aromatic and polar residues. To fully characterize the ligand-binding sites of CBMs, polar residues in the vicinity of the predicted HARs in the *in silico* structures may also be involved in ligand-binding function.

**Table 3:** Occurrence times for residues that flank known ligand-binding aromatic residues.

G	N	S	T	D	A	Q	I	E	K	V	L	Y	P	C	M	F	H	R	W
49	43	34	34	33	30	27	22	22	20	16	13	9	8	6	6	6	4	3	3

The occurrence times were derived from the two upstream and downstream residues flanking 97 known aromatic ligand-binding residues and were transformed into weighted scores for HAR prediction.

### How to use DS-CBMs

**Figure 7:**
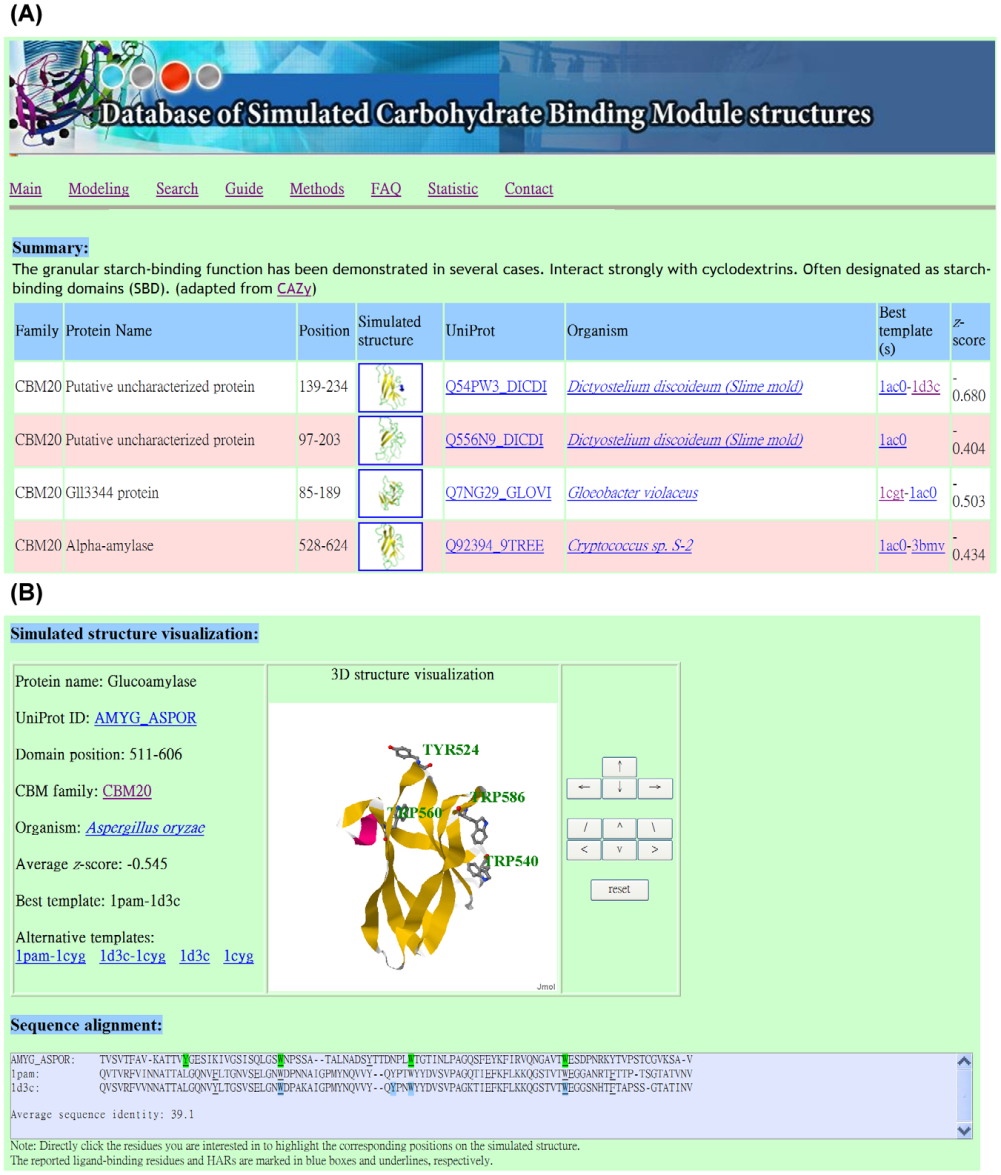
Snapshots of DS-CBMs system. (A) The DS-CBMs search results for the CBM20 family. The CBM entry, organism, and structure template(s) are accessible *via* cross-database hyperlinks. (B) Simulated 3D structure visualization for *Aspergillus oryzae* glucoamylase (CBM20). The CBM profiles, 3D structure manipulation, HAR identification and known ligand-binding residue annotation are provided.

All *in silico* structures simulated in this study have been deposited into DS-CBMs at http://dscbm.life.nthu.edu.tw and http://dscbm.cs.ntou.edu.tw. The 817 target CBMs can be searched for by their CBM family, protein, or organism names, and by keywords. In answer to a query, matched CBMs with brief profiles as shown in Figure 7(A) are returned. Additionally, the CBM entry, organism, and structure template(s) are accessible *via* cross-database hyperlinks. Finally, the *in silico* structure with the best surface-potential *z* score is identified. Directly clicking on the structural preview image allows users to switch into the interactive 3D visualization interface where the structure can be manipulated as shown in Figure 7(B). Rotation and shift functions are provided in the control panel and all operating functions can be accessed by clicking the right mouse button. At the bottom, HARs and known ligand-binding residues are annotated in the sequence view. Finally, clicking on a specific amino acid within the target sequence highlights its position in the corresponding *in silico* structure. Researchers may also submit a CBM sequence to the on-line structure modeling system. When the modeling procedure is completed, a page similar to that shown in Figure 7(B) will be generated.

### Conclusion

We developed an automated and generalized homology modeling procedure for CBMs. A total of 817 *in silico* structures with the minimum *z* scores were generated and deposited in the DS-CBMs. The major challenge of CBM homology model building is that the target and template sequences contain only a small number of homologues, i.e., low sequence identity, so that conventional homology modeling procedures may fail to build reliable *in silico* structures. Our main contribution has been to improve target-template sequence alignment by incorporating the conserved positions of secondary structure elements and HARs into the FIA algorithm, which is used to provide accurate target-template alignment for homology model building. Additionally, using a single template to build the model may not be the best strategy. The template filter step was incorporated to discover multiple homologous templates for model building and double-template-based structure model building conducted lower surface-potential *z* scores for *in silico* structures. Finally, low surface-potential *z* scores and assessment of the *in silico* structures suggest that the structures likely have been “correctly” built and are functionally relevant. HAR identification demonstrated its higher true positive rate and lower false positive rate for liagnd-binding aromatic residue prediction for CBMs. In conclusion, more than 95% of the CBMs do not have solved structures, and the ligand specificity of a particular CBM is mainly determined by the positions and orientations of the aromatic ligand-binding residues. The *in silico* CBM structures can be integrated with current databases like CAZy and Pfam to discover potential ligand-binding residues. The integrated *in silico* and *in vitro* resources would facilitate the comprehension of functional similarities and diversities among all CBMs.

## Materials and Methods

### System definition and overview

**Figure 8:**
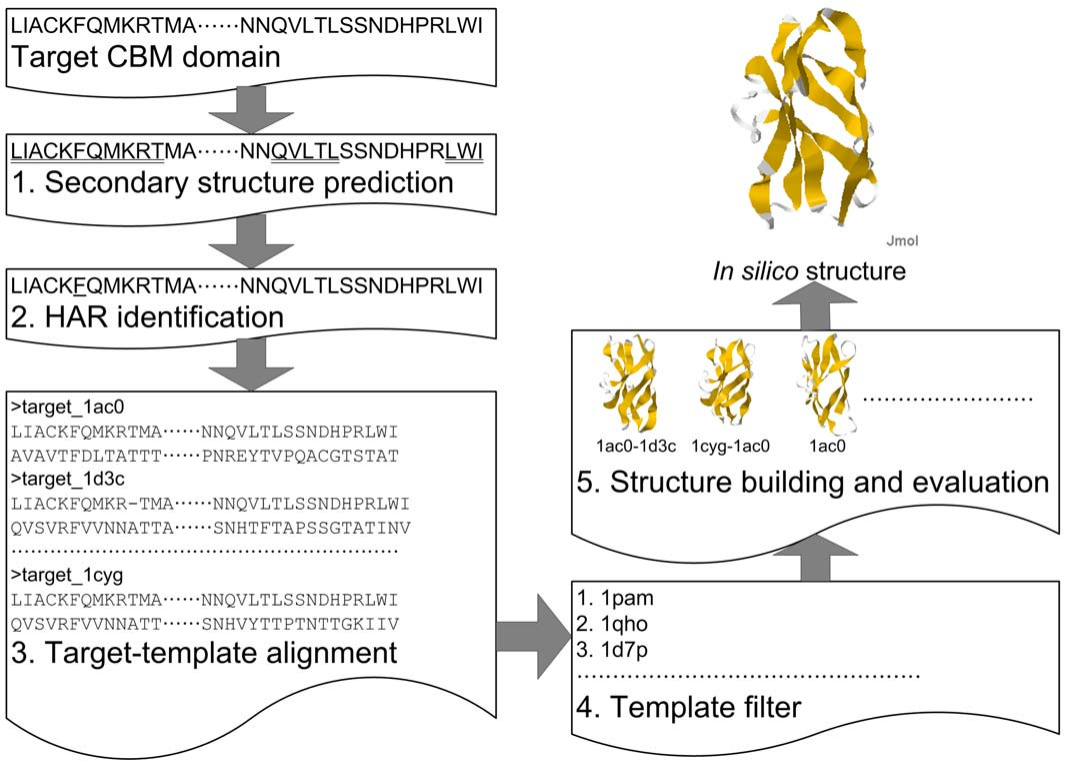
Flowchart for FIA-based homology modeling. The proposed structure modeling procedure for CBMs comprises of five modules: secondary structure prediction, hydrophilic aromatic residue (HAR) identification, target-template alignment, template filter, and structure building and evaluation. Details are described in Materials and Methods.

The objective of this study was to build accurate *in silico* structures for CBMs without manual intervention. Figure 8 illustrated the flowchart for the modeling procedure and the mathematical definitions are defined as follows. Given a target CBM sequence of *t* and a set of *m* non-redundant CBM templates denoted *P*, the goal was to generate the most reliable *in silico* structure s* for *t* by identifying the minimum surface-potential *z* score after the sequence of *t* was considered each of the templates in *P*. Secondary structure prediction and identification of hydrophilic aromatic residues (HARs) were performed in the first two steps. For the third step, a set of pairwise alignments denoted *A* between a sequence of *t* and each of the template sequences in *P* was made using feature-incorporated alignment (FIA). For the fourth step, the set of candidate target-template alignments denoted *CA* was ranked from the top *k-*matched template among *A* according to the proposed identity level and similarity level. Subsequently, a *CSS* set was build that contained the candidate *in silico* structures of *t* derived from single- and double-template alignments in the *CA*. s* was identified as the *in silico* structure of *t* in the *CSS* that had the minimum surface-potential *z* score. The implementation of each step is detailed in the following sections.

### Secondary structure prediction

CBM domains have been classified into seven fold families, i.e., β-sandwiches, β-trefoils, cysteine knots, unique, OB folds, hevein folds, and hevein-like folds [8]. The β-sandwich and β-trefoil folds have been found in CBMs, and these β-strand folds are conserved among CBM families. We first predicted the secondary structure elements of the CBMs using the Discrimination of protein Secondary structure Class (DSC) algorithm [27]. When a residue was predicted to be α-helical or β-stranded with a probability of <50%, it was annotated as a loop residue (Only high confident predictions for helix and strand were labeled for latter sequence alignment.). On average, a three-state (helix, strand and loop) accuracy of 70.1% was obtained by DSC.

### Hydrophilic aromatic residue identification

CBM ligand-binding sites are of three types: surface binding (type A), glycan-chain binding (type B), and small-sugar binding (type C) [7]. Type A CBMs possess a platform-like hydrophobic surface consisted of aromatic residues. In contrast, the binding site architecture of type B CBMs shapes a cleft or groove arrangement in which aromatic residues interact with free single polysaccharide chains. Due to stereo restriction in the binding site, Type C CBMs lacks of the cleft form as in type B CBMs only bind mono-, di-, or trisaccharides. In general, CBM ligand-binding sites contain aromatic and polar residues. In terms of their ligand-binding interactions, aromatic residues are involved in aromatic stacking, whereas polar residues form hydrogen bonds with ligands [8]. Additionally, polar residues adjacent to aromatic residues can enlarge the surface area of neighboring regions thereby increasing the contact area(s) between the binding residues and ligands. In our previous study, the number of preferred occurrence time that polar residues flanked an aromatic residue was determined for starch-binding CBMs [21], and the aromatic residues that were flanked on both sides by polar residues were defined as HARs. For this study, the occurrence time for two upstream and downstream amino acids that flank 97 known ligand-binding aromatic residues was summarized in Table 3 (the structure template profiles including known ligand-binding aromatic residues are shown in Supporting Information Table S2). Interestingly, the preferred occurrence times for Asn (N), Ser (S), Asp (D), Thr (T), Gln (Q), Glu (E), and Lys (K) are observed and consistent with their ligand-binding functionality for polar residues in CBMs. To identify potential HARs, the weighted scores (directly derived from the occurrence times) of the sets of two upstream and downstream flanking residues for each aromatic residue were summed. When the sum was greater than or equal to the cut-off threshold of 97 (see Discussion), these aromatic residues were defined as HARs. For example, the motif GSWNP had a score of 134 (49 + 34 + 43 + 8), and therefore, the central tryptophan was identified as an HAR. Conversely, the motif PTYKA had a score of 92 (8 + 34 + 20 + 30), and the central tyrosine was therefore not identified as an HAR. Step 1 and step 2 identified the core-conserved sequence signatures associated with secondary structure prediction and HARs that were then used for sequence alignment.

### Target-template alignment by FIA

To evaluate the target-template sequence matching, a target sequence *t* was aligned one by one with the templates in *P* as described by Equation 1, where FIA represents the feature-incorporated alignment [21], and *A* is the set of preliminary alignments used in the template filter step.
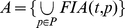
(1)


The FIA algorithm adopted the affine gap-penalty model and the Blosum62 matrix [28], [29]. The individual residues of a full-length CBM were not uniformly weighted by the FIA, which emphasized alignment of the secondary structure elements and conserved HAR positions.

### Template filter

Usually, conventional template selection that is used during homology modeling attempts to identify the best template for subsequent *in silico* modeling. However, the criteria used for sequence matching may conflict with that used for structure building and assessment, e.g., the surface-potential *z* score for the modeled target structure. Therefore, we used a template filter that had been designed to select templates according to the proposed identity level and similarity level for *t*' and *p*' as defined in Equation 2 and Equation 3, respectively. Here, *ident*(*t*', *p*'), *sim*(*t*', *p*'), and *gap*(*t*', *p*') represent the number of aligned identical residues, similar residues, and opening gaps, respectively, in *t*' and *p*', where *t*' and *p*' denote the sequences of *t* and *p* aligned using FIA. The proposed identity level (similarity level) charged extra opening penalties. The larger the identity level (similarity level) is, the larger the sequence homology is and the fewer gaps present. Subsequently, Equation 4 was used to filter homologue target-template sequence alignments for *CA*, where *top_il*(*A*) and *top_sl*(*A*) represent the top *k* target-template alignments in *A* for the identity level and similarity level, respectively. In practice, between *k* and 2*k* target-template alignments were contained in *CA* for use in model building.
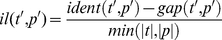
(2)

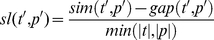
(3)

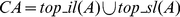
(4)


### Structure model building and evaluation

With the use of the template filter, *CA* was obtained to build the *CSS* for the sequence of *t*. Intuitively, multiple-template-based homology modeling would be expected to improve the accuracy of a modeled structure, but that is not always the case. Larsson reported that double-template and triple-template modeling was more accurate than was considering four or more templates [30]. Multiple-template-based manner here refers to model construction based on the integration of multiple templates instead of averaging individual models from individual templates. To ensure the quality of the model and the efficiency of the process, we incorporated only single and combinations of double templates into the modeling process. We did not include triple templates because their computational costs were extremely high. The *CSS* were generated using single templates, combinations of double templates and Equation 5 where *s_modeling*(*a*) and *d_modeling*(*b*, *c*) are for modeling using a single template, *a*, and double templates, *b* and *c*, respectively. For quality assessment, the quality of each *in silico* structure was evaluated according to its surface-potential *z* score. The most reliable structure s* in *CSS* was chosen as the one that had the lowest average surface-potential *z* score as determined by Equation 6, where *z_*score(*s*) is the *z* score for candidate simulated structure *s*.
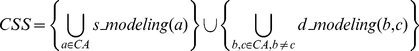
(5)

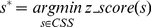
(6)


### Materials

CBM with domain sizes exceeding 85 residues in length were identified in the Pfam database and grouped according to their CBM classification [31]. A total of 93 non-redundant templates were then selected from 18 CBM families. Of these, 3, 2, 4, 11, 22, 2, 9, 3, 1, 25, 1, 5, 3, and 2 were from the CBM families CBM2 (PF00553), CBM3 (PF00942), CBM4&9&16&22 (PF02018), CBM6 (PF03422), CBM13 (PF00652), CBM17&28 (PF03424), CBM20 (PF00686), CBM21 (PF03370), CBM25 (PF03423), CBM32 (PF00754), CBM33 (PF03067), CBM34 (PF02903), CBM40 (PF02973), and CBM51 (PF08305), respectively. The criteria for their selection were as follows. If multiple crystal structures were available for a CBM, the one with the best resolution was used. If more than one structure of the same CBM had the same resolution, which was also the best, the one with the smaller *R*-factor was used. For the ensemble of NMR solution structures for a CBM, which were used when a corresponding crystal structure was unavailable, the structure used was randomly selected from the ensemble. A total of 817 representative CBM sequences were used as targets to predict their *in silico* structures. The targets all had pairwise sequence identities below 80% and were from 19 CBM families among which 15, 25, 45, 36, 92, 1, 4, 97, 78, 33, 174, 131, 39, 4, and 43 were selected, respectively, from CBM2, CBM3, CBM4&9&16&22, CBM6, CBM13, CBM15 (PF03426), CBM17&28, CBM20, CBM21, CBM25, CBM32, CBM33, CBM34, CBM40, and CBM51.

The alignment performance for the 817 target sequences against the 93 template sequences was compared among FIA and six alignment tools, MUSCLE v3.8.31 [32], ClustalW2 v2.1 [33], DIALIGN-TX v1.0.2 [34], T-COFFEE v5.31 [35], ProbCons v1.12 [36], and MAFFT v6.850 [37] with default parameter settings used. The structure model building engine was Modeller v9.8. The 3D visualization and the preview images were rendered by Jmol (http://www.jmol.org/) and PyMol (http://www.pymol.org/), respectively. Surface-potential *z* scores were calculated by PROSA2003 [38].

## Supporting Information

Figure S1 **CBMs containing aromatic residues conserved to known ligand-binding residues and their** ***in silico*** **structures.** Sixteen CBMs without *in vitro* structures are predicted. O30421, *Caldocellum saccharolyticum* xylanase; O30426, *Caldocellum saccharolyticum* xylanase; O88043, *Streptomyces coelicolor* putative secreted arabinosidase; Q60043, *Thermoanaero bacterium* endoxylanase; O69822, *Streptomyces coelicolor* putative secreted protein; Q9KBL8, *Bacillus halodurans* glucan 1,4-β-glucosidase; Q1ENB1, *Guillardia theta* putative starch binding domain protein; Q6R608, *Solanum tuberosum* 4-α-glucanotransferase; PPR3C, *Danio rerio* protein phosphatase 1 regulatory subunit 3C; Q89ZX7, *Bacteroides thetaiotaomicron* putative uncharacterized protein; Q8LEV3, *Arabidopsis thaliana* putative uncharacterized protein; Q9U5D0, *Drosophila melanogaster* hemolectin; Q82M60, *Streptomyces avermitilis* putative secreted protein; A8GAL3, *Serratia proteamaculans* α-amylase catalytic region; Q8A1R7, *Bacteroides thetaiotaomicron* α-galactosidase; and Q9XGC0, *Vigna unguiculata* starch synthase isoform SS III. Known ligand-binding aromatic residues are highlighted in ball and stick model. HARs and non-HARs are texted in green and blue, respectively. The 3D structures were rendered by Jmol (http://www.jmol.org/).
(TIF)

Table S1 **Profile summaries for CBMs containing aromatic residues conserved to reported ligand-binding residues.** Sixteen CBMs with target-template sequence identity less than 30% are selected for prediction analysis. The HARs highlighted italics denote aromatic residues conserved to known ligand-binding residues in corresponding template(s). No experimental data concerning their ligand-binding abilities is available for the unannotated HARs.
(DOC)

Table S2 **Profiles for non-redundant CBM structure templates.** 93 non-redundant structures are selected as templates. The HARs with bold fonts indicate experimentally determined ligand-binding residues. No experimental data concerning their ligand-binding abilities is available for the unannotated HARs.
(DOC)
