# Supplementary figures and images for: Hydrophilic Aromatic Residue and in silico Structure for Carbohydrate Binding Module
Source: PLoS One. 2011 Sep 22;6(9):e24814. doi: 10.1371/journal.pone.0024814 (PMC3178555; doi:10.1371/journal.pone.0024814)

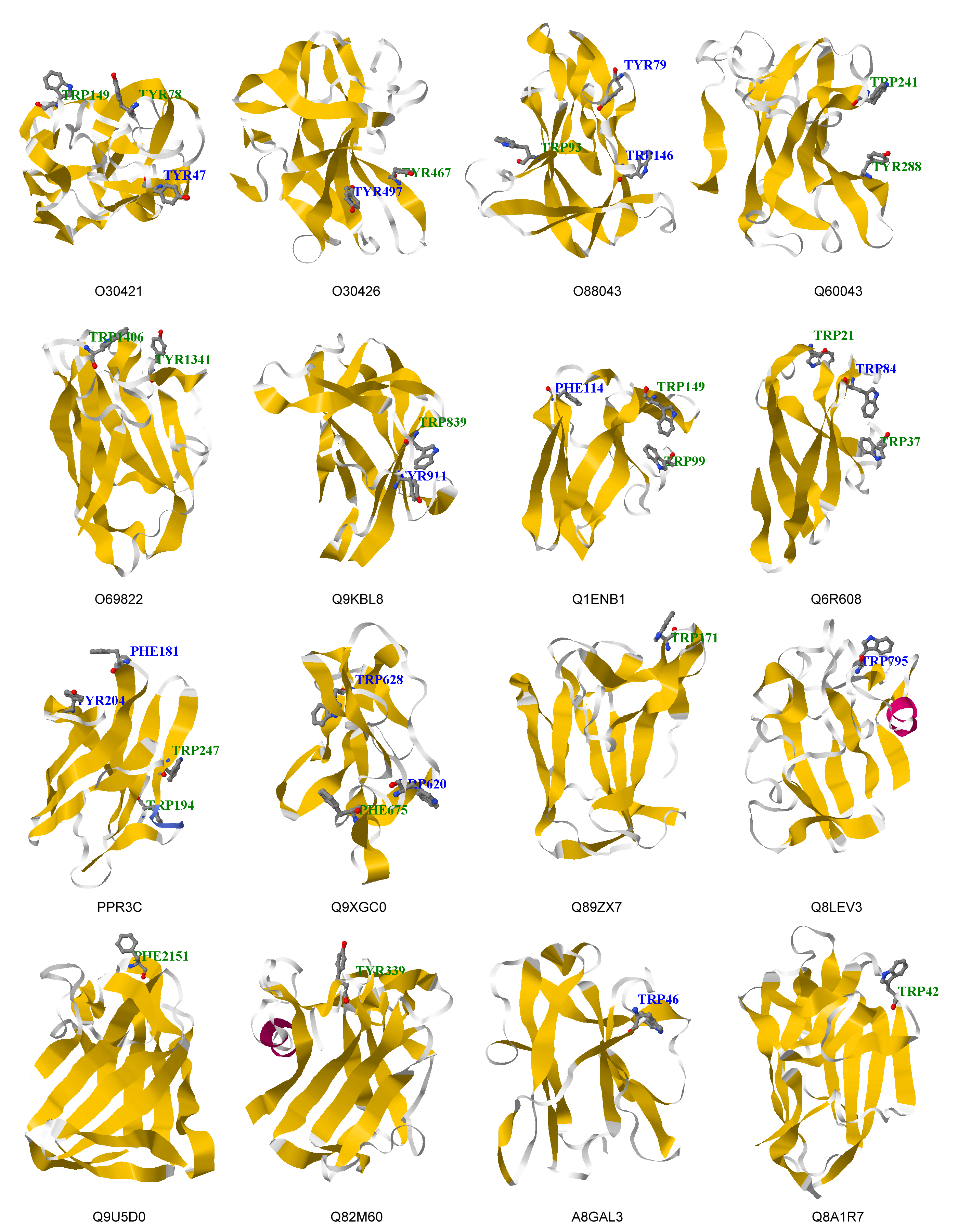

Supplement: Figure S1 — CBMs containing aromatic residues conserved to known ligand-binding residues and their in silico structures. Sixteen CBMs without in vitro structures are predicted. O30421, Caldocellum saccharolyticum xylanase; O30426, Caldocellum saccharolyticum xylanase; O88043, Streptomyces coelicolor putative secreted arabinosidase; Q60043, Thermoanaero bacterium endoxylanase; O69822, Streptomyces coelicolor putative secreted protein; Q9KBL8, Bacillus halodurans glucan 1,4-β-glucosidase; Q1ENB1, Guillardia theta putative starch binding domain protein; Q6R608, Solanum tuberosum 4-α-glucanotransferase; PPR3C, Danio rerio protein phosphatase 1 regulatory subunit 3C; Q89ZX7, Bacteroides thetaiotaomicron putative uncharacterized protein; Q8LEV3, Arabidopsis thaliana putative uncharacterized protein; Q9U5D0, Drosophila melanogaster hemolectin; Q82M60, Streptomyces avermitilis putative secreted protein; A8GAL3, Serratia proteamaculans α-amylase catalytic region; Q8A1R7, Bacteroides thetaiotaomicron α-galactosidase; and Q9XGC0, Vigna unguiculata starch synthase isoform SS III. Known ligand-binding aromatic residues are highlighted in ball and stick model. HARs and non-HARs are texted in green and blue, respectively. The 3D structures were rendered by Jmol (http://www.jmol.org/). (TIF) [file pone.0024814.s001.tif]
