# Supplementary material for: Hydrophilic Aromatic Residue and in silico Structure for Carbohydrate Binding Module
Source: PLoS One. 2011 Sep 22;6(9):e24814. doi: 10.1371/journal.pone.0024814 (PMC3178555; doi:10.1371/journal.pone.0024814)
Supplement: Table S1 — Profile summaries for CBMs containing aromatic residues conserved to reported ligand-binding residues. Sixteen CBMs with target-template sequence identity less than 30% are selected for prediction analysis. The HARs highlighted italics denote aromatic residues conserved to known ligand-binding residues in corresponding template(s). No experimental data concerning their ligand-binding abilities is available for the unannotated HARs. (DOC) [file pone.0024814.s002.doc]

Table S1. Profile summaries for CBMs containing aromatic residues conserved to reported ligand-binding residues.

| Family | UniProt | Protein | Position | Organism | HARs | Template(s) | Identity | *Z* score | Ref(s) |
| --- | --- | --- | --- | --- | --- | --- | --- | --- | --- |
| CBM4 | O30421 | Xylanase | 41–177 | *Caldocellum saccharolyticum* | F49, *Y78*, *W149*, Y159 | 2j1v–1qho | 28.3 | –0.227 | [1,2] |
| CBM4 | O30426 | Xylanase | 376–514 | *Caldocellum saccharolyticum* | F384, W391, *Y467*, F474, W480, F487 | 1dyo | 25.7 | –0.461 | [3] |
| CBM4 | O88043 | Putative secreted arabinosidase | 69–201 | *Streptomyces coelicolor* | Y87, *W93*, Y129, W178, F185 | 1gui–1heh | 29.7 | –0.276 | [4,5] |
| CBM4 | Q60043 | Endoxylanase | 194–329 | *Thermoanaero bacterium* | F202, W210, Y222, Y230, *W241*, *Y288*, F294, W302, F309, Y316 | 1dyo–1nbc | 29.9 | –0.414 | [3,6] |
| CBM6 | O69822 | Putative secreted protein | 1311–1451 | *Streptomyces coelicolor* | F1313, W1328, *Y1341*, W1345, Y1348, F1353, Y1365, F1385, Y1394, *W1406* | 1w9s–1od3 | 29.6 | –0.594 | [7,8] |
| CBM6 | Q9KBL8 | Glucan 1,4-β-glucosidase | 809–926 | *Bacillus halodurans* | Y813, Y816, *W839*, W893, F914, F918 | 1od3–3c7f | 28.4 | –0.630 | [8,9] |
| CBM20 | Q1ENB1 | Putative starch binding domain protein | 65–168 | *Guillardia theta* | *W99*, F117, F122, *W149* | 1d3c–1pam | 26.8 | –0.732 | [10,11] |
| CBM20 | Q6R608 | 4-α-glucanotransferase | 8–104 | *Solanum tuberosum* | *W21*, *W37*, W56, Y66, Y70 | 1ac0–1cyg | 25.8 | –0.641 | [12] |
| CBM21 | PPR3C | Protein phosphatase 1 regulatory subunit 3C | 152–258 | *Danio rerio* | F191, *W194*, Y204, Y209, F217, Y239, *W247* | 2v8l | 25.2 | –0.148 | [13] |
| CBM32 | Q89ZX7 | Putative uncharacterized protein | 139–266 | *Bacteroides thetaiotaomicron* | Y155, F159, *W171*, F205, Y211, Y221, Y227, F240, F260 | 2v5d–1w8o | 29.7 | –0.765 | [14,15] |
| CBM32 | Q8LEV3 | Putative uncharacterized protein | 665–802 | *Arabidopsis thaliana* | F675, Y686, F691, F736, W745, F763 | 2w1s–3eyp | 24.6 | –0.240 | [16] |
| CBM32 | Q9U5D0 | Hemolectin | 2092–2222 | *Drosophila melanogaster* | W2119, F2134, *F2151*, Y2154, F2182 | 3bn6–2vm9 | 28.2 | –0.170 | [17,18] |
| CBM32 | Q82M60 | Putative secreted protein | 195–344 | *Streptomyces avermitilis* | Y199, F202, W218, F222, W229, W233, Y260, Y264, F337, *Y339* | 2jda–1k3i | 27.9 | –0.271 | [19,20] |
| CBM34 | A8GAL3 | α-amylase catalytic region | 1–108 | *Serratia proteamaculans* | W5, W88, W94, F105 | 1ea9–1j0h | 28.7 | –0.366 | [21,22] |
| CBM51 | Q8A1R7 | α-galactosidase | 22–163 | *Bacteroides thetaiotaomicron* | Y37, *W42*, W156, F161 | 2vmh | 26.8 | –0.276 | [23] |
| CBM53 | Q9XGC0 | Starch synthase isoform SS III | 599–688 | *Vigna unguiculata* | F605, F625, *F675*, F679 | 1ac0–1knm | 22.2 | –0.419 | [24] |

Sixteen CBMs with target-template sequence identity less than 30% are selected for prediction analysis. The HARs with itatics denote aromatic residues conserved to known ligand-binding residues in corresponding template(s). No experimental data concerning their ligand-binding abilities is available for the unannotated HARs.

**References**

1. Boraston AB, Wang D, Burke RD (2006) Blood group antigen recognition by a Streptococcus pneumoniae virulence factor. J Biol Chem 281: 35263-35271.

2. Dauter Z, Dauter M, Brzozowski AM, Christensen S, Borchert TV, et al. (1999) X-ray structure of Novamyl, the five-domain "maltogenic" alpha-amylase from Bacillus stearothermophilus: maltose and acarbose complexes at 1.7A resolution. Biochemistry 38: 8385-8392.

3. Charnock SJ, Bolam DN, Turkenburg JP, Gilbert HJ, Ferreira LM, et al. (2000) The X6 "thermostabilizing" domains of xylanases are carbohydrate-binding modules: structure and biochemistry of the Clostridium thermocellum X6b domain. Biochemistry 39: 5013-5021.

4. Boraston AB, Nurizzo D, Notenboom V, Ducros V, Rose DR, et al. (2002) Differential oligosaccharide recognition by evolutionarily-related beta-1,4 and beta-1,3 glucan-binding modules. J Mol Biol 319: 1143-1156.

5. Bolam DN, Xie H, White P, Simpson PJ, Hancock SM, et al. (2001) Evidence for synergy between family 2b carbohydrate binding modules in Cellulomonas fimi xylanase 11A. Biochemistry 40: 2468-2477.

6. Tormo J, Lamed R, Chirino AJ, Morag E, Bayer EA, et al. (1996) Crystal structure of a bacterial family-III cellulose-binding domain: a general mechanism for attachment to cellulose. EMBO J 15: 5739-5751.

7. van Bueren AL, Morland C, Gilbert HJ, Boraston AB (2005) Family 6 carbohydrate binding modules recognize the non-reducing end of beta-1,3-linked glucans by presenting a unique ligand binding surface. J Biol Chem 280: 530-537.

8. Boraston AB, Notenboom V, Warren RA, Kilburn DG, Rose DR, et al. (2003) Structure and ligand binding of carbohydrate-binding module CsCBM6-3 reveals similarities with fucose-specific lectins and "galactose-binding" domains. J Mol Biol 327: 659-669.

9. Vandermarliere E, Bourgois TM, Winn MD, van Campenhout S, Volckaert G, et al. (2009) Structural analysis of a glycoside hydrolase family 43 arabinoxylan arabinofuranohydrolase in complex with xylotetraose reveals a different binding mechanism compared with other members of the same family. Biochem J 418: 39-47.

10. Uitdehaag JC, Kalk KH, van Der Veen BA, Dijkhuizen L, Dijkstra BW (1999) The cyclization mechanism of cyclodextrin glycosyltransferase (CGTase) as revealed by a gamma-cyclodextrin-CGTase complex at 1.8-A resolution. J Biol Chem 274: 34868-34876.

11. Harata K, Haga K, Nakamura A, Aoyagi M, Yamane K (1996) X-ray structure of cyclodextrin glucanotransferase from alkalophilic Bacillus sp. 1011. Comparison of two independent molecules at 1.8 A resolution. Acta Crystallogr D Biol Crystallogr 52: 1136-1145.

12. Sorimachi K, Le Gal-Coeffet MF, Williamson G, Archer DB, Williamson MP (1997) Solution structure of the granular starch binding domain of Aspergillus niger glucoamylase bound to beta-cyclodextrin. Structure 5: 647-661.

13. Tung JY, Chang MD, Chou WI, Liu YY, Yeh YH, et al. (2008) Crystal structures of the starch-binding domain from Rhizopus oryzae glucoamylase reveal a polysaccharide-binding path. Biochem J 416: 27-36.

14. Ficko-Blean E, Gregg KJ, Adams JJ, Hehemann JH, Czjzek M, et al. (2009) Portrait of an enzyme, a complete structural analysis of a multimodular {beta}-N-acetylglucosaminidase from Clostridium perfringens. J Biol Chem 284: 9876-9884.

15. Watson JN, Newstead S, Dookhun V, Taylor G, Bennet AJ (2004) Contribution of the active site aspartic acid to catalysis in the bacterial neuraminidase from Micromonospora viridifaciens. FEBS Lett 577: 265-269.

16. Ficko-Blean E, Boraston AB (2009) N-acetylglucosamine recognition by a family 32 carbohydrate-binding module from Clostridium perfringens NagH. J Mol Biol 390: 208-220.

17. Shao C, Novakovic VA, Head JF, Seaton BA, Gilbert GE (2008) Crystal structure of lactadherin C2 domain at 1.7A resolution with mutational and computational analyses of its membrane-binding motif. J Biol Chem 283: 7230-7241.

18. Aragao KS, Satre M, Imberty A, Varrot A (2008) Structure determination of Discoidin II from Dictyostelium discoideum and carbohydrate binding properties of the lectin domain. Proteins 73: 43-52.

19. Abbott DW, Hrynuik S, Boraston AB (2007) Identification and characterization of a novel periplasmic polygalacturonic acid binding protein from Yersinia enterolitica. J Mol Biol 367: 1023-1033.

20. Firbank SJ, Rogers MS, Wilmot CM, Dooley DM, Halcrow MA, et al. (2001) Crystal structure of the precursor of galactose oxidase: an unusual self-processing enzyme. Proc Natl Acad Sci U S A 98: 12932-12937.

21. Hondoh H, Kuriki T, Matsuura Y (2003) Three-dimensional structure and substrate binding of Bacillus stearothermophilus neopullulanase. J Mol Biol 326: 177-188.

22. Lee HS, Kim MS, Cho HS, Kim JI, Kim TJ, et al. (2002) Cyclomaltodextrinase, neopullulanase, and maltogenic amylase are nearly indistinguishable from each other. J Biol Chem 277: 21891-21897.

23. Gregg KJ, Finn R, Abbott DW, Boraston AB (2008) Divergent modes of glycan recognition by a new family of carbohydrate-binding modules. J Biol Chem 283: 12604-12613.

24. Notenboom V, Boraston AB, Williams SJ, Kilburn DG, Rose DR (2002) High-resolution crystal structures of the lectin-like xylan binding domain from Streptomyces lividans xylanase 10A with bound substrates reveal a novel mode of xylan binding. Biochemistry 41: 4246-4254.
