# Supplementary material for: Hydrophilic Aromatic Residue and in silico Structure for Carbohydrate Binding Module
Source: PLoS One. 2011 Sep 22;6(9):e24814. doi: 10.1371/journal.pone.0024814 (PMC3178555; doi:10.1371/journal.pone.0024814)
Supplement: Table S2 — Profiles for non-redundant CBM structure templates. 93 non-redundant structures are selected as templates. The HARs with bold fonts indicate experimentally determined ligand-binding residues. No experimental data concerning their ligand-binding abilities is available for the unannotated HARs. (DOC) [file pone.0024814.s003.doc]

Table S2. Profiles for non-redundant CBM structure templates.

| Family | PDB | Chain | Position | HARs | Ligand(s) | Reference(s) |
| --- | --- | --- | --- | --- | --- | --- |
| CBM2 | 1exg | A | 8–107 | W17, F21, W38, **W54**, **W72**, F84, F86, F100 | Cellohexaose, [β-D-glucopyranosyl-(1,4)]_5_-D-glucose | [1] |
| CBM2 | 2cwr | A | 265–358 | **W274**, Y280, Y290, F307, **W308**, Y318, **W326**, F334 | Chitin | [2] |
| CBM2 | 1heh | C | 560–644 | **W570**, Y578, W586, **W602**, F624, Y629 | Oat spelt xylan, xylohexaose | [3] |
| CBM3 | 1nbc | A | 4–95 | Y7, **Y67**, F78, Y91 | Crystalline cellulose | [4] |
| CBM3 | 1g43 | A | 8–98 | F10, Y64, **Y70**, F81 | Crystalline cellulose | [5] |
| CBM4 | 1gu3 | A | 3–130 | W16, **Y19**, **Y43**, Y60, **Y85**, F106, Y112 | Cellopentaose | [6] |
| CBM4 | 2zex | A | 4–133 | F12, W19, **W22**, Y62, W67, F70, F78, Y93, F100, W105, F118, **W127** | Mannopentaose, ellopentaose | [7] |
| CBM4 | 1dyo | A | 8–143 | F15, W22, Y38, **W53**, F79, **Y103**, | Xylooligosaccharides, xylan | [8] |
| CBM4 | 1gui | A | 12–146 | **Y32**, Y42, Y48, F65, W68, Y72, Y77, F81, W102, Y105, F106, W116, F119 | Cellopentaose | [6] |
| CBM6 | 2w47 | A | 8–131 | Y15, Y26, Y31, Y34, Y41, W44, Y54, **W91**, W94, F129 | Ecuronic acid, δ4,5-anhydrogalaturonic acid | [9] |
| CBM6 | 1uxz | A | 7–129 | **Y33**, W39, Y42, Y55, **W92**, W95, F111, W120 | Xylohexaose, cellohexaose | [10] |
| CBM6 | 1uxx | X | 8–128 | Y12, **Y34**, Y40, F48, F55, **W92**, Y95, F115 | Xylohexaose, cellohexaose | [10] |
| CBM6 | 2w87 | A | 16–138 | F33, F38, Y67, Y89, **W99**, W102 | Uronic acid sugars, Ecuronic acid, δ4,5-anhydrogalacturonic acid | [9] |
| CBM6 | 2vzp | A | 7–127 | Y25, F30, Y33, Y53, F84, **W90**, W93 | Uronic acid sugars, Ecuronic acid, δ4,5-anhydrogalaturonic acid | [9] |
| CBM6 | 2cdp | A | 8–138 | F12, F19, **Y30**, **Y40**, Y46, Y49, Y59, Y63, W84, **W97**, F100, Y109, **W127**, F134 | Agarose | [11] |
| CBM6 | 1w9s | A | 16–140 | Y20, **W42**, W48, **W99**, W102, Y116, F122 | Xylobiose, aminarihexaose | [12] |
| CBM6 | 2dck | A | 213–327 | Y223, F231, Y237, F245, F256, F282, F284, Y288, Y292, W317, Y320 | *N.A.* | *N.A.* |
| CBM6 | 1od3 | A | 30–148 | Y34, Y38, **Y56**, Y61, Y65, F70, Y105, **F112**, Y115, F135 | Xylotriose, cellobiose, laminaribiose | [13] |
| CBM6 | 3c7f | A | 359–486 | W365, **W394**, F402, F468, F477 | Xylotetraose | [14] |
| CBM6 | 1w0n | A | 16–131 | Y26, F34, **Y40**, Y45, Y48, Y51, F52, F85, F87, **W120**, Y123 | Xylotriose | [15] |
| CBM13 | 1abr | B | 12–136 | Y29, Y33, Y72, Y79, W98 | Galactose | [16] |
| CBM13 | 2q3n | B | 12–136 | Y33, Y72, Y79, Y94, W95, W98 | Galactose | [17] |
| CBM13 | 1rzo | B | 2007–2131 | F2029, W2045, W2093, Y2125 | β-D-galactose | *N.A.* |
| CBM13 | 2ffu | A | 446–563 | F463 | UDP-*N*-acetylgalactosamine | [18] |
| CBM13 | 2d7i | A | 459–587 | W497 | *N*-acetylgalactosamine-Ser | [19] |
| CBM13 | 1qxm | A | 156–281 | F179, Y190, Y197, Y217, Y221, W231, Y235, Y258, Y273 | Galactose | [20] |
| CBM13 | 1m2t | B | 255–379 | F276, Y315, Y317, W338, W341 | Adenine | [21] |
| CBM13 | 1ce7 | B | 4–128 | F25, Y64, Y66, W87, **W90** | Galactose, nucleotide, *N*-acetyl-D-glucosamine | [21] |
| CBM13 | 1dqg | A | 2–114 | Y9, W64, Y78, F89, Y100, Y111 | Galactose | [22] |
| CBM13 | 3c9z | A | 1–123 | F3, Y21, F43, Y117 | Galactose | [23] |
| CBM13 | 2zqo | A | 131–255 | **W161**, F189 | Galactose, lactose | [24] |
| CBM13 | 2vse | A | 298–435 | Y302, Y320, Y328, F331, F333, W338, Y342, Y349, W363, Y377, Y386, F396 | *N.A.* | [25] |
| CBM13 | 1ybi | A | 161–288 | W190, F204, F217, Y250, **Y265**, Y280 | Lactose | [26] |
| CBM13 | 2d1z | A | 311–433 | W350, Y352, Y361, W383 | *N.A.* | *N.A.* |
| CBM13 | 1vcl | A | 105–232 | Y134 | Galactose | [27] |
| CBM13 | 2vlc | A | 292–416 | Y313, Y354, Y359, W378, Y410 | Mannose, xylopyranose | [28] |
| CBM13 | 1hwm | B | 9–131 | Y29, Y52, Y74 | Galactose | [29] |
| CBM13 | 2aai | B | 7–131 | F28, Y67, Y69, W93, Y125 | *N.A.* | [30] |
| CBM13 | 1knm | A | 5–127 | W44, Y55, W77 | Lactose | [31] |
| CBM13 | 1xhb | A | 430–580 | F457, Y470 | *N*-acetylgalactosamine | [32] |
| CBM13 | 1xez | A | 460–572 | F468, Y503, Y545, Y562 | β-octyl glucoside | [33] |
| CBM13 | 1ggp | B | 12–136 | F24, Y79, W91, Y126 | *N.A.* | *N.A.* |
| CBM17 | 1uww | A | 7–189 | F22, W30, W32, **W68**, Y88, Y118, **W119**, Y126, F130, F170, F177 | Non-crystalline cellulose | [34] |
| CBM17 | 1j83 | A | 1027–1205 | F1034, F1038, F1041, F1049, F1087, W1088, W1098, Y1105, W1135, F1149, Y1157, F1171, F1189 | Cellotetraose | [35] |
| CBM20 | 1vem | A | 419–515 | Y438, W449, W467, W495, W502 | Starch, glycogen | [36] |
| CBM20 | 1ac0 | A | 514–610 | **Y527**, **W543**, **Y556** | Starch | [37] |
| CBM20 | 1qho | A | 581–682 | **W612**, **W662** | Maltose, acarbose derived hexasaccharide | [38] |
| CBM20 | 1cgt | A | 584–681 | Y603, W614, F625, W661,F669 | *N.A.* | [39] |
| CBM20 | 1d3c | A | 586–682 | Y605, **W616**, **W662** | Maltotetraose | [40] |
| CBM20 | 1pam | A | 586–682 | F605, W616, W662, F670 | *N.A.* | [41] |
| CBM20 | 3bmv | A | 583–679 | Y597, Y602, W613, W659 | Heptasaccharide analogue | [42] |
| CBM20 | 1cqy | A | 419–515 | Y438, W449, W467, W495, W502 | *N.A.* | *N.A.* |
| CBM20 | 1cyg | A | 579–676 | Y598, W609, W656 | *N.A.* | *N.A.* |
| CBM21 | 2v8l | A | 3–105 | Y14, Y16, F21, **Y32**, **W47**, **F58**, Y67, F72, **Y94**, Y102 | β-cyclodextrin | [43] |
| CBM21 | 2eef | A | 30–136 | F69, W72, Y75, Y87, F95, Y124, W125 | *N.A.* | *N.A.* |
| CBM21 | 2djm | A | 3–105 | Y14, Y16, F21, **Y32**, **W47**, F58, Y67, F72, Y94, Y102 | β-cyclodextrin, maltoheptaose | [44] |
| CBM25 | 2c3w | A | 9–87 | W20, **W34**, Y47, Y50, F67, **W74**, F84 | α-glucooligosaccharides, granular starch | [45] |
| CBM32 | 2jda | A | 9–131 | Y18, W38, W45, F76, Y77, W84, F118, **W125** | Polygalacturonic acid | [46] |
| CBM32 | 2vm9 | A | 17–151 | Y24, F37, F46, W52, F62, W87, Y91, W102, Y105, F125, Y141 | *N.A.* | [47] |
| CBM32 | 1sdd | B | 1881–2017 | F1887, Y1904, W1907, F1916, F1977, Y2008 | Copper ion, *N*-acetyl-D-glucosamine, 2-(acetylamino)-2-deoxy-A-D-glucopyranose | [48] |
| CBM32 | 1czt | A | 16–153 | F22, W26, W27, Y30, W47, Y88, F113, F126, F127, W143 | PLS molecules | [49] |
| CBM32 | 1d7p | M | 2189–2323 | Y2195, F2196, **F2200**, W2203, F2283, F2290 | Membrane | [50] |
| CBM32 | 1k3i | A | 13–143 | Y43, W81, W96, W105, F106, Y113, F116, W137 | α-D-glucose | [51] |
| CBM32 | 3bn6 | A | 16–155 | Y22, Y23, **W26**, **F31**, F46, W49, Y94, W101, Y104, F115, F129, F133 | Membrane | [52] |
| CBM32 | 2w1s | A | 809–941 | W816, Y819, F850, W879, W893, F915, **W935**, F938 | *N*-acetylglucosamine, galacturonate polymers | [53] |
| CBM32 | 1w8o | A | 512–641 | Y583, Y586, W594, F603 | α-lactose | [54] |
| CBM32 | 2qqi | A | 290–421 | Y297, W301, W315, W369, F381, W411 | Heparin | [55] |
| CBM32 | 2orz | A | 290–421 | Y297, W301, W315, W369, F381, F392, W411 | Heparin | [56] |
| CBM32 | 2qqj | A | 292–424 | Y299, W318, Y356, W414 | Heparin | [55] |
| CBM32 | 2vcc | A | 34–150 | W58, Y64, Y75, F83, Y95, F103, Y108 | Heparin | [57] |
| CBM32 | 3cqo | A | 159–285 | **F166**, Y172, **W183**, **F220**, Y268 | α-L-fucose | [58] |
| CBM32 | 3gza | A | 348–453 | W413 | *N.A.* | *N.A.* |
| CBM32 | 3eyp | A | 354–472 | Y378, F379, W418, W429 | *N.A.* | *N.A.* |
| CBM32 | 3ggl | A | 303–432 | W335, F377, W386, F392, F394 | *N.A.* | *N.A.* |
| CBM32 | 2j1v | A | 22–145 | **Y31**, **F47**, F92, Y97 | β-L-fucose | [59] |
| CBM32 | 2z4f | A | 45–182 | **W52**, Y60, Y122, W132, Y148 | Collagen | [60] |
| CBM32 | 1k12 | A | 16–148 | Y46, Y69 | Fucose | [61] |
| CBM32 | 2v5d | A | 630–763 | F647, W661, Y680, Y701, F712, F731, F757 | *N*-acetyllactosamine | [62] |
| CBM32 | 3f2z | A | 303–432 | W335, W386, F392, F394 | *N.A.* | *N.A.* |
| CBM32 | 2v72 | A | 6–134 | **W40**, F75, Y79, W97, F108, F128 | Galactose, *N*-acetylgalactosamine | [63] |
| CBM32 | 1tvg | A | 15–132 | W39, F85, F122, F125 | Galactose | [64] |
| CBM32 | 2j7m | A | 630–763 | F647, **W661**, Y680, Y701, F712, F731, **F757** | (β-D-galactosyl-1,4-β-D-*N*-acetylglucosamine), galactose and the type II blood group H-trisaccharide | [65] |
| CBM33 | 2bem | A | 28–194 | F65, W94, F106, W128, F139, F147, W178, F187 | β-chitin | [66] |
| CBM34 | 1ji1 | A | 11–125 | F13, F20, F39, F74, W77, W102, Y103, F118 | α-CD, β-CD | [67] |
| CBM34 | 1wzl | A | 1–117 | Y14, Y45, Y95, F101, F111 | *N.A.* | *N.A.* |
| CBM34 | 1j0h | A | 1-121 | F14, Y18, W47, W52, Y89 | Panose, maltotetraose, isopanose | [68] |
| CBM34 | 1sma | A | 1-121 | F14, Y18, F41, W52, F68 | β-CD | [69] |
| CBM34 | 1ea9 | C | 1-117 | F14, Y18 | β-CD | [70] |
| CBM40 | 2jkb | A | 42–228 | Y48, F88, F97, Y101, F102, W134, Y160, F211 | IT-sialidase, 2,7-anhydro-Neu5Ac, α2-3 linked sialic acid substrates, the carboxylate of the sialic acid substrate | [71] |
| CBM40 | 2sli | A | 81–275 | Y100, Y123, F135, F170, Y184, F248, Y258 | Neu5Ac analogues | [72] |
| CBM40 | 2v73 | A | 1–187 | Y11, F32, Y66, F67, Y70, Y78, F89, F119, F135, F145, Y155, F166, Y170, Y178 | Galactose | [63] |
| CBM51 | 2vmh | A | 904–1050 | Y909, **Y922**, Y948, F976, F1002, F1044 | Methyl-β-D-galactose | [73] |
| CBM51 | 2vng | A | 37–205 | W49, Y108, Y121, **Y135**, Y151, F157, Y163, Y186, **W192**, F203 | Trisaccharide | [73] |

93 non-redundant structures are selected as templates. The HARs with bold font indicate experimentally determined ligand-binding residues. No experimental data concerning their ligand-binding abilities is available for the unannotated HARs.

**References**

1. Xu GY, Ong E, Gilkes NR, Kilburn DG, Muhandiram DR, et al. (1995) Solution structure of a cellulose-binding domain from Cellulomonas fimi by nuclear magnetic resonance spectroscopy. Biochemistry 34: 6993-7009.

2. Nakamura T, Mine S, Hagihara Y, Ishikawa K, Ikegami T, et al. (2008) Tertiary structure and carbohydrate recognition by the chitin-binding domain of a hyperthermophilic chitinase from Pyrococcus furiosus. J Mol Biol 381: 670-680.

3. Bolam DN, Xie H, White P, Simpson PJ, Hancock SM, et al. (2001) Evidence for synergy between family 2b carbohydrate binding modules in Cellulomonas fimi xylanase 11A. Biochemistry 40: 2468-2477.

4. Tormo J, Lamed R, Chirino AJ, Morag E, Bayer EA, et al. (1996) Crystal structure of a bacterial family-III cellulose-binding domain: a general mechanism for attachment to cellulose. EMBO J 15: 5739-5751.

5. Shimon LJ, Pages S, Belaich A, Belaich JP, Bayer EA, et al. (2000) Structure of a family IIIa scaffoldin CBD from the cellulosome of Clostridium cellulolyticum at 2.2 A resolution. Acta Crystallogr D Biol Crystallogr 56: 1560-1568.

6. Boraston AB, Nurizzo D, Notenboom V, Ducros V, Rose DR, et al. (2002) Differential oligosaccharide recognition by evolutionarily-related beta-1,4 and beta-1,3 glucan-binding modules. J Mol Biol 319: 1143-1156.

7. Bae B, Ohene-Adjei S, Kocherginskaya S, Mackie RI, Spies MA, et al. (2008) Molecular basis for the selectivity and specificity of ligand recognition by the family 16 carbohydrate-binding modules from Thermoanaerobacterium polysaccharolyticum ManA. J Biol Chem 283: 12415-12425.

8. Charnock SJ, Bolam DN, Turkenburg JP, Gilbert HJ, Ferreira LM, et al. (2000) The X6 "thermostabilizing" domains of xylanases are carbohydrate-binding modules: structure and biochemistry of the Clostridium thermocellum X6b domain. Biochemistry 39: 5013-5021.

9. Montanier C, van Bueren AL, Dumon C, Flint JE, Correia MA, et al. (2009) Evidence that family 35 carbohydrate binding modules display conserved specificity but divergent function. Proc Natl Acad Sci U S A 106: 3065-3070.

10. Pires VM, Henshaw JL, Prates JA, Bolam DN, Ferreira LM, et al. (2004) The crystal structure of the family 6 carbohydrate binding module from Cellvibrio mixtus endoglucanase 5a in complex with oligosaccharides reveals two distinct binding sites with different ligand specificities. J Biol Chem 279: 21560-21568.

11. Henshaw J, Horne-Bitschy A, van Bueren AL, Money VA, Bolam DN, et al. (2006) Family 6 carbohydrate binding modules in beta-agarases display exquisite selectivity for the non-reducing termini of agarose chains. J Biol Chem 281: 17099-17107.

12. van Bueren AL, Morland C, Gilbert HJ, Boraston AB (2005) Family 6 carbohydrate binding modules recognize the non-reducing end of beta-1,3-linked glucans by presenting a unique ligand binding surface. J Biol Chem 280: 530-537.

13. Boraston AB, Notenboom V, Warren RA, Kilburn DG, Rose DR, et al. (2003) Structure and ligand binding of carbohydrate-binding module CsCBM6-3 reveals similarities with fucose-specific lectins and "galactose-binding" domains. J Mol Biol 327: 659-669.

14. Vandermarliere E, Bourgois TM, Winn MD, van Campenhout S, Volckaert G, et al. (2009) Structural analysis of a glycoside hydrolase family 43 arabinoxylan arabinofuranohydrolase in complex with xylotetraose reveals a different binding mechanism compared with other members of the same family. Biochem J 418: 39-47.

15. Jamal-Talabani S, Boraston AB, Turkenburg JP, Tarbouriech N, Ducros VM, et al. (2004) Ab initio structure determination and functional characterization of CBM36; a new family of calcium-dependent carbohydrate binding modules. Structure 12: 1177-1187.

16. Tahirov TH, Lu TH, Liaw YC, Chen YL, Lin JY (1995) Crystal structure of abrin-a at 2.14 A. J Mol Biol 250: 354-367.

17. Bagaria A, Surendranath K, Ramagopal UA, Ramakumar S, Karande AA (2006) Structure-function analysis and insights into the reduced toxicity of Abrus precatorius agglutinin I in relation to abrin. J Biol Chem 281: 34465-34474.

18. Fritz TA, Raman J, Tabak LA (2006) Dynamic association between the catalytic and lectin domains of human UDP-GalNAc:polypeptide alpha-N-acetylgalactosaminyltransferase-2. J Biol Chem 281: 8613-8619.

19. Kubota T, Shiba T, Sugioka S, Furukawa S, Sawaki H, et al. (2006) Structural basis of carbohydrate transfer activity by human UDP-GalNAc: polypeptide alpha-N-acetylgalactosaminyltransferase (pp-GalNAc-T10). J Mol Biol 359: 708-727.

20. Inoue K, Sobhany M, Transue TR, Oguma K, Pedersen LC, et al. (2003) Structural analysis by X-ray crystallography and calorimetry of a haemagglutinin component (HA1) of the progenitor toxin from Clostridium botulinum. Microbiology 149: 3361-3370.

21. Krauspenhaar R, Rypniewski W, Kalkura N, Moore K, DeLucas L, et al. (2002) Crystallisation under microgravity of mistletoe lectin I from Viscum album with adenine monophosphate and the crystal structure at 1.9 A resolution. Acta Crystallogr D Biol Crystallogr 58: 1704-1707.

22. Liu Y, Chirino AJ, Misulovin Z, Leteux C, Feizi T, et al. (2000) Crystal structure of the cysteine-rich domain of mannose receptor complexed with a sulfated carbohydrate ligand. J Exp Med 191: 1105-1116.

23. Maveyraud L, Niwa H, Guillet V, Svergun DI, Konarev PV, et al. (2009) Structural basis for sugar recognition, including the Tn carcinoma antigen, by the lectin SNA-II from Sambucus nigra. Proteins 75: 89-103.

24. Suzuki R, Kuno A, Hasegawa T, Hirabayashi J, Kasai KI, et al. (2009) Sugar-complex structures of the C-half domain of the galactose-binding lectin EW29 from the earthworm Lumbricus terrestris. Acta Crystallogr D Biol Crystallogr 65: 49-57.

25. Treiber N, Reinert DJ, Carpusca I, Aktories K, Schulz GE (2008) Structure and mode of action of a mosquitocidal holotoxin. J Mol Biol 381: 150-159.

26. Arndt JW, Gu J, Jaroszewski L, Schwarzenbacher R, Hanson MA, et al. (2005) The structure of the neurotoxin-associated protein HA33/A from Clostridium botulinum suggests a reoccurring beta-trefoil fold in the progenitor toxin complex. J Mol Biol 346: 1083-1093.

27. Uchida T, Yamasaki T, Eto S, Sugawara H, Kurisu G, et al. (2004) Crystal structure of the hemolytic lectin CEL-III isolated from the marine invertebrate Cucumaria echinata: implications of domain structure for its membrane pore-formation mechanism. J Biol Chem 279: 37133-37141.

28. Azzi A, Wang T, Zhu DW, Zou YS, Liu WY, et al. (2009) Crystal structure of native cinnamomin isoform III and its comparison with other ribosome inactivating proteins. Proteins 74: 250-255.

29. Pascal JM, Day PJ, Monzingo AF, Ernst SR, Robertus JD, et al. (2001) 2.8-A crystal structure of a nontoxic type-II ribosome-inactivating protein, ebulin l. Proteins 43: 319-326.

30. Rutenber E, Katzin BJ, Ernst S, Collins EJ, Mlsna D, et al. (1991) Crystallographic refinement of ricin to 2.5 A. Proteins 10: 240-250.

31. Notenboom V, Boraston AB, Williams SJ, Kilburn DG, Rose DR (2002) High-resolution crystal structures of the lectin-like xylan binding domain from Streptomyces lividans xylanase 10A with bound substrates reveal a novel mode of xylan binding. Biochemistry 41: 4246-4254.

32. Fritz TA, Hurley JH, Trinh LB, Shiloach J, Tabak LA (2004) The beginnings of mucin biosynthesis: the crystal structure of UDP-GalNAc:polypeptide alpha-N-acetylgalactosaminyltransferase-T1. Proc Natl Acad Sci U S A 101: 15307-15312.

33. Olson R, Gouaux E (2005) Crystal structure of the Vibrio cholerae cytolysin (VCC) pro-toxin and its assembly into a heptameric transmembrane pore. J Mol Biol 350: 997-1016.

34. Jamal S, Nurizzo D, Boraston AB, Davies GJ (2004) X-ray crystal structure of a non-crystalline cellulose-specific carbohydrate-binding module: CBM28. J Mol Biol 339: 253-258.

35. Notenboom V, Boraston AB, Chiu P, Freelove AC, Kilburn DG, et al. (2001) Recognition of cello-oligosaccharides by a family 17 carbohydrate-binding module: an X-ray crystallographic, thermodynamic and mutagenic study. J Mol Biol 314: 797-806.

36. Hirata A, Adachi M, Utsumi S, Mikami B (2004) Engineering of the pH optimum of Bacillus cereus beta-amylase: conversion of the pH optimum from a bacterial type to a higher-plant type. Biochemistry 43: 12523-12531.

37. Sorimachi K, Le Gal-Coeffet MF, Williamson G, Archer DB, Williamson MP (1997) Solution structure of the granular starch binding domain of Aspergillus niger glucoamylase bound to beta-cyclodextrin. Structure 5: 647-661.

38. Dauter Z, Dauter M, Brzozowski AM, Christensen S, Borchert TV, et al. (1999) X-ray structure of Novamyl, the five-domain "maltogenic" alpha-amylase from Bacillus stearothermophilus: maltose and acarbose complexes at 1.7A resolution. Biochemistry 38: 8385-8392.

39. Klein C, Schulz GE (1991) Structure of cyclodextrin glycosyltransferase refined at 2.0 A resolution. J Mol Biol 217: 737-750.

40. Uitdehaag JC, Kalk KH, van Der Veen BA, Dijkhuizen L, Dijkstra BW (1999) The cyclization mechanism of cyclodextrin glycosyltransferase (CGTase) as revealed by a gamma-cyclodextrin-CGTase complex at 1.8-A resolution. J Biol Chem 274: 34868-34876.

41. Harata K, Haga K, Nakamura A, Aoyagi M, Yamane K (1996) X-ray structure of cyclodextrin glucanotransferase from alkalophilic Bacillus sp. 1011. Comparison of two independent molecules at 1.8 A resolution. Acta Crystallogr D Biol Crystallogr 52: 1136-1145.

42. Kelly RM, Leemhuis H, Rozeboom HJ, van Oosterwijk N, Dijkstra BW, et al. (2008) Elimination of competing hydrolysis and coupling side reactions of a cyclodextrin glucanotransferase by directed evolution. Biochem J 413: 517-525.

43. Tung JY, Chang MD, Chou WI, Liu YY, Yeh YH, et al. (2008) Crystal structures of the starch-binding domain from Rhizopus oryzae glucoamylase reveal a polysaccharide-binding path. Biochem J 416: 27-36.

44. Liu YN, Lai YT, Chou WI, Chang MD, Lyu PC (2007) Solution structure of family 21 carbohydrate-binding module from Rhizopus oryzae glucoamylase. Biochem J 403: 21-30.

45. Boraston AB, Healey M, Klassen J, Ficko-Blean E, Lammerts van Bueren A, et al. (2006) A structural and functional analysis of alpha-glucan recognition by family 25 and 26 carbohydrate-binding modules reveals a conserved mode of starch recognition. J Biol Chem 281: 587-598.

46. Abbott DW, Hrynuik S, Boraston AB (2007) Identification and characterization of a novel periplasmic polygalacturonic acid binding protein from Yersinia enterolitica. J Mol Biol 367: 1023-1033.

47. Aragao KS, Satre M, Imberty A, Varrot A (2008) Structure determination of Discoidin II from Dictyostelium discoideum and carbohydrate binding properties of the lectin domain. Proteins 73: 43-52.

48. Adams TE, Hockin MF, Mann KG, Everse SJ (2004) The crystal structure of activated protein C-inactivated bovine factor Va: Implications for cofactor function. Proc Natl Acad Sci U S A 101: 8918-8923.

49. Macedo-Ribeiro S, Bode W, Huber R, Quinn-Allen MA, Kim SW, et al. (1999) Crystal structures of the membrane-binding C2 domain of human coagulation factor V. Nature 402: 434-439.

50. Pratt KP, Shen BW, Takeshima K, Davie EW, Fujikawa K, et al. (1999) Structure of the C2 domain of human factor VIII at 1.5 A resolution. Nature 402: 439-442.

51. Firbank SJ, Rogers MS, Wilmot CM, Dooley DM, Halcrow MA, et al. (2001) Crystal structure of the precursor of galactose oxidase: an unusual self-processing enzyme. Proc Natl Acad Sci U S A 98: 12932-12937.

52. Shao C, Novakovic VA, Head JF, Seaton BA, Gilbert GE (2008) Crystal structure of lactadherin C2 domain at 1.7A resolution with mutational and computational analyses of its membrane-binding motif. J Biol Chem 283: 7230-7241.

53. Ficko-Blean E, Boraston AB (2009) N-acetylglucosamine recognition by a family 32 carbohydrate-binding module from Clostridium perfringens NagH. J Mol Biol 390: 208-220.

54. Watson JN, Newstead S, Dookhun V, Taylor G, Bennet AJ (2004) Contribution of the active site aspartic acid to catalysis in the bacterial neuraminidase from Micromonospora viridifaciens. FEBS Lett 577: 265-269.

55. Appleton BA, Wu P, Maloney J, Yin J, Liang WC, et al. (2007) Structural studies of neuropilin/antibody complexes provide insights into semaphorin and VEGF binding. EMBO J 26: 4902-4912.

56. Vander Kooi CW, Jusino MA, Perman B, Neau DB, Bellamy HD, et al. (2007) Structural basis for ligand and heparin binding to neuropilin B domains. Proc Natl Acad Sci U S A 104: 6152-6157.

57. Ficko-Blean E, Stubbs KA, Nemirovsky O, Vocadlo DJ, Boraston AB (2008) Structural and mechanistic insight into the basis of mucopolysaccharidosis IIIB. Proc Natl Acad Sci U S A 105: 6560-6565.

58. Bianchet MA, Odom EW, Vasta GR, Amzel LM (2010) Structure and specificity of a binary tandem domain F-lectin from striped bass (Morone saxatilis). J Mol Biol 401: 239-252.

59. Boraston AB, Wang D, Burke RD (2006) Blood group antigen recognition by a Streptococcus pneumoniae virulence factor. J Biol Chem 281: 35263-35271.

60. Ichikawa O, Osawa M, Nishida N, Goshima N, Nomura N, et al. (2007) Structural basis of the collagen-binding mode of discoidin domain receptor 2. EMBO J 26: 4168-4176.

61. Bianchet MA, Odom EW, Vasta GR, Amzel LM (2002) A novel fucose recognition fold involved in innate immunity. Nat Struct Biol 9: 628-634.

62. Ficko-Blean E, Gregg KJ, Adams JJ, Hehemann JH, Czjzek M, et al. (2009) Portrait of an enzyme, a complete structural analysis of a multimodular {beta}-N-acetylglucosaminidase from Clostridium perfringens. J Biol Chem 284: 9876-9884.

63. Boraston AB, Ficko-Blean E, Healey M (2007) Carbohydrate recognition by a large sialidase toxin from Clostridium perfringens. Biochemistry 46: 11352-11360.

64. Ramelot TA, Raman S, Kuzin AP, Xiao R, Ma LC, et al. (2009) Improving NMR protein structure quality by Rosetta refinement: a molecular replacement study. Proteins 75: 147-167.

65. Ficko-Blean E, Boraston AB (2006) The interaction of a carbohydrate-binding module from a Clostridium perfringens N-acetyl-beta-hexosaminidase with its carbohydrate receptor. J Biol Chem 281: 37748-37757.

66. Vaaje-Kolstad G, Houston DR, Riemen AH, Eijsink VG, van Aalten DM (2005) Crystal structure and binding properties of the Serratia marcescens chitin-binding protein CBP21. J Biol Chem 280: 11313-11319.

67. Kamitori S, Abe A, Ohtaki A, Kaji A, Tonozuka T, et al. (2002) Crystal structures and structural comparison of Thermoactinomyces vulgaris R-47 alpha-amylase 1 (TVAI) at 1.6 A resolution and alpha-amylase 2 (TVAII) at 2.3 A resolution. J Mol Biol 318: 443-453.

68. Hondoh H, Kuriki T, Matsuura Y (2003) Three-dimensional structure and substrate binding of Bacillus stearothermophilus neopullulanase. J Mol Biol 326: 177-188.

69. Kim JS, Cha SS, Kim HJ, Kim TJ, Ha NC, et al. (1999) Crystal structure of a maltogenic amylase provides insights into a catalytic versatility. J Biol Chem 274: 26279-26286.

70. Lee HS, Kim MS, Cho HS, Kim JI, Kim TJ, et al. (2002) Cyclomaltodextrinase, neopullulanase, and maltogenic amylase are nearly indistinguishable from each other. J Biol Chem 277: 21891-21897.

71. Gut H, King SJ, Walsh MA (2008) Structural and functional studies of Streptococcus pneumoniae neuraminidase B: An intramolecular trans-sialidase. FEBS Lett 582: 3348-3352.

72. Luo Y, Li SC, Li YT, Luo M (1999) The 1.8 A structures of leech intramolecular trans-sialidase complexes: evidence of its enzymatic mechanism. J Mol Biol 285: 323-332.

73. Gregg KJ, Finn R, Abbott DW, Boraston AB (2008) Divergent modes of glycan recognition by a new family of carbohydrate-binding modules. J Biol Chem 283: 12604-12613.
